# Illumination Intelligent Adaptation and Analysis Framework: A comprehensive solution for enhancing nighttime driving fatigue monitoring

**DOI:** 10.1371/journal.pone.0308201

**Published:** 2024-08-14

**Authors:** Zenghui Tian, Nur Safinas Albakry, Yinghui Du

**Affiliations:** Faculty of Art, Sustainability & Creative Industry, Sultan Idris Education University, Tanjung Malim, Perak, Malaysia; University of Lagos Faculty of Engineering, NIGERIA

## Abstract

Nighttime driving presents a critical challenge to road safety due to insufficient lighting and increased risk of driver fatigue. Existing methods for monitoring driver fatigue, mainly focusing on behavioral analysis and biometric monitoring, face significant challenges under low-light conditions. Their effectiveness, especially in dynamic lighting environments, is limited by their dependency on specific environmental conditions and active driver participation, leading to reduced accuracy and practicality in real-world scenarios. This study introduces a novel ‘Illumination Intelligent Adaptation and Analysis Framework (IIAAF)’, aimed at addressing these limitations and enhancing the accuracy and practicality of driver fatigue monitoring under nighttime low-light conditions. The IIAAF framework employs a multidimensional technology integration, including comprehensive body posture analysis and facial fatigue feature detection, per-pixel dynamic illumination adjustment technology, and a light variation feature learning system based on Convolutional Neural Networks (CNN) and time-series analysis. Through this integrated approach, the framework is capable of accurately capturing subtle fatigue signals in nighttime driving environments and adapting in real-time to rapid changes in lighting conditions. Experimental results on two independent datasets indicate that the IIAAF framework significantly improves the accuracy of fatigue detection under nighttime low-light conditions. This breakthrough not only enhances the effectiveness of driving assistance systems but also provides reliable scientific support for reducing the risk of accidents caused by fatigued driving. These research findings have significant theoretical and practical implications for advancing intelligent driving assistance technology and improving nighttime road safety.

## 1 Introduction

### 1.1 Background

Driving at night is like driving in a horror movie, with only the moon and stars to navigate by. As night falls, the road becomes a stage filled with unknowns and dangers. In the development of intelligent driving assistance systems, driver fatigue monitoring has become a key technology and an ethical imperative to ensure road safety. The development of this technology is not only a protection for driving safety but also deeply reflects respect for human life dignity. For instance, studies show that driver fatigue is associated with 16% to 20% of serious accidents on highways in the UK, Australia, and Brazil [1]. Fatigued driving is particularly dangerous at night, as drivers begin to feel fatigued after only 3.06 hours of nighttime driving [2]. Moreover, Rezaee et al. (2022) pointed out that road accidents caused by fatigued driving account for 35–45%, causing 1,550 deaths and 71,000 injuries annually, with an economic loss of up to 12.5 billion USD [3].

Existing fatigue detection technologies are like navigating a modern city with an ancient map, limited in effectiveness. These techniques mainly focus on traditional behavioral analysis and biometric monitoring, but their effectiveness, especially under dynamic lighting conditions at night, remains to be improved. The development of computer vision and machine learning technologies in recent years, like a light in the darkness, has brought new possibilities for nighttime driving safety. In particular, image processing technologies based on dynamic contrast adjustment have shown potential in processing low-light video data. The incorporation of emotion recognition technologies, especially in terms of illumination intensity grading, offers a new perspective for a comprehensive understanding of the driver’s mental and physical state. However, a significant problem emerges when attempting to integrate these technologies for real-world applications: their efficacy in actual traffic environments, especially under varied and unpredictable night-time lighting conditions, has not been sufficiently explored. This gap in application highlights a critical issue: the need for a robust and adaptable solution capable of functioning reliably in the diverse lighting scenarios encountered during night-time driving.

The development of this technology is not just a matter of enhancing road safety; it reflects a profound respect for human life and dignity. Fatigued driving is especially perilous at night, with drivers beginning to experience fatigue after relatively short periods, leading to a high incidence of serious accidents. The economic and human costs are staggering, underscoring the urgent need for more effective fatigue detection methods.

This paper addresses this pressing issue by proposing an innovative approach that leverages recent advancements in computer vision and machine learning. The goal is to transcend the limitations of existing technologies by integrating advanced image processing, which is adept at handling low-light video data, and emotion recognition technologies for a more nuanced understanding of the driver’s condition. The proposed “Illumination Intelligent Adaptation and Analysis Framework” (IIAAF) marks a significant step forward in nighttime driver fatigue detection. It is specifically designed to tackle the challenges posed by low-light conditions, offering a more comprehensive and adaptive solution. By doing so, the research aims to significantly enhance the effectiveness of driver assistance systems and, ultimately, contribute to safer nighttime driving experiences.

### 1.2 Literature review

In recent years, significant progress has been made in nighttime driver fatigue detection technology, yet there are still some limitations. You et al. (2017) proposed a new eye detection algorithm and eye verification process, improving accuracy and robustness [4]. Ma et al. (2017) introduced a dual-stream CNN system based on deep video, achieving an accuracy rate of 91.57% in detecting nighttime driver fatigue, surpassing the baseline system of the latest technology [5].

According to Sikander and Anwar (2018), fatigue detection technologies include subjective reporting, driver biometrics, physical characteristics of the driver, vehicle characteristics during driving, and a combination of these features [6]. Matthews et al. (2019) suggested that advancements in nighttime driver fatigue detection technology include spectral frequency analysis of electrocardiograms (ECG) and eye gaze duration, as well as developing algorithms adapted to the driver’s sleep history [7]. Doudou et al. (2020) emphasized that drowsiness detection technologies for drivers include measurements based on the vehicle, driver behavior, and physiological signals, but there are issues in terms of invasiveness, accuracy, and practical use [8]. Karuppusamy and Kang (2020) proposed hybrid multimodal system uses EEG, gyroscope, and image processing, with a detection accuracy rate of 93.91% in identifying driver fatigue [9].

Furthermore, Bakker et al. (2021) proposed a multi-stage, multi-feature machine learning approach that accurately detects driver drowsiness under natural road driving conditions, potentially reducing road accidents related to drowsiness [10]. Recent research by Yogesh et al. (2022) shows that a driver drowsiness detection and alert system using the YOLO algorithm can identify fatigued drivers by analyzing facial features and eye detection, issuing alerts when the driver appears drowsy [11].

Although significant progress has been made in recent years in nighttime driver fatigue detection technology, existing technologies still show inadequacies in terms of non-invasiveness, real-time capabilities, environmental adaptability, and comprehensiveness. Especially under low-light conditions, these limitations become more apparent. Current technologies often rely on specific environmental conditions and active participation of the driver, which may lead to reduced detection accuracy and practicality in real-world applications. The research aims to enhance the accuracy and practicality of nighttime driver fatigue monitoring under low-light conditions by combining posture estimation, per-pixel dynamic illumination adjustment technology, and a light variation feature learning system.

### 1.3 Research contributions

This study aims to improve the accuracy and practicality of nighttime driver fatigue monitoring, especially under low-light conditions. The research contributions are focused on the following three key technological innovations:

- **Fatigue Dynamic Analysis Based on Posture Estimation:** The approach is capable of accurately identifying facial signs of fatigue, such as yawning, and also analyzes overall changes in the driver’s body posture, such as slumping shoulders or leaning forward. This comprehensive monitoring approach can capture subtle fatigue signals that may appear after prolonged driving, offering a more holistic and precise fatigue detection than traditional facial analysis.
- **Per-Pixel Dynamic Illumination Adjustment Technology:** The research proposes a per-pixel dynamic illumination adjustment technology, employing advanced image processing algorithms for meticulous brightness and contrast adjustments on each pixel. This technique adapts in real-time to fluctuations in lighting conditions during nighttime driving, such as flickering streetlights or headlights from oncoming vehicles, ensuring clear visibility of the driver’s facial features under complex lighting conditions.
- **Light Variation Feature Learning System:** By combining CNN and time-series analysis models, the system is capable of learning and predicting light variation patterns from historical image data. This learning and prediction mechanism enables the system to adapt more intelligently and accurately under the constantly changing lighting conditions of nighttime driving, thereby enhancing the stability and accuracy of fatigue detection.

These innovations together constitute the proposed “Illumination Intelligent Adaptation and Analysis Framework (IIAAF),” offering a comprehensive and innovative solution for driver fatigue detection under low-light conditions at night. The research provides significant theoretical and technical foundations for developing more efficient driving assistance systems.

## 2 Proposed approach

### 2.1 Problem description

**Fig 1:**
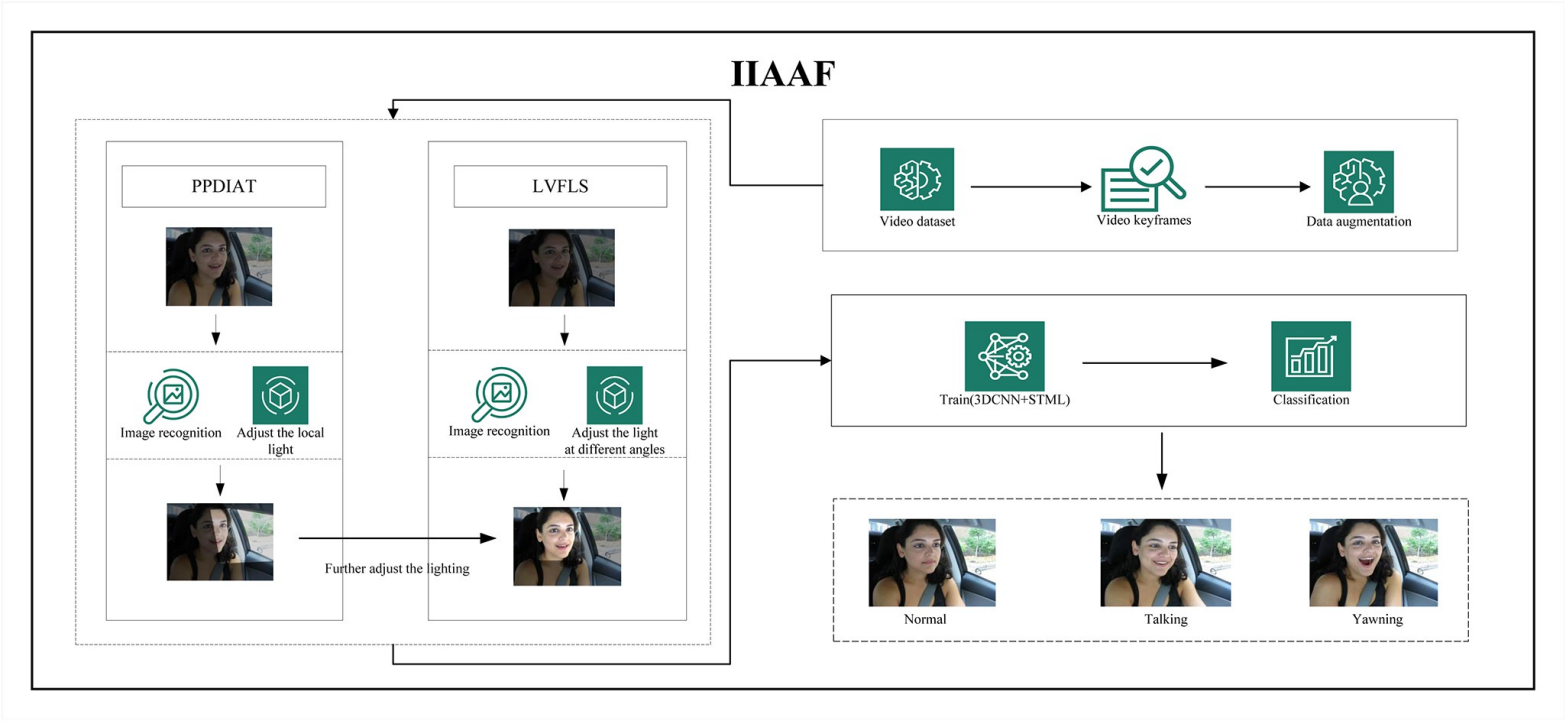
IIAAF overall framework (Image source: Yawning detection dataset (DOI: https://dx.doi.org/10.21227/e1qm-hb90), licensed under CC BY 4.0.).

In intelligent driving assistance systems, accurately analyzing a driver’s facial behavior (such as yawning) from continuous video frames to identify fatigue states is a challenging task. Facial behaviors are significant indicators of fatigue states, as they are often unconscious and can be effectively captured by automated systems. The goal is to design an algorithm capable of accurately identifying fatigue patterns from video frames captured under low brightness and uneven lighting conditions. The overall framework is shown in Fig 1.

The experimental steps in the above framework include video data collection and processing. Image recognition technology is used to adjust local lighting and lighting at different angles to improve image recognizability. The study performs data augmentation. It trains a model that combines three-dimensional convolutional neural networks (3DCNN) and spatiotemporal long short-term memory networks (STML). This model captures spatiotemporal features in video frames, accurately classifying the driver’s facial behaviors.

Specifically, the mathematical problem faced can be described as:

$$\begin{array}{c} F\left(X_{t}\right)=\sum_{i=1}^{N}w_{i}\cdot g\left(h\left(X_{t-i},\theta \right),\phi \right)+\sum_{j=1}^{M}v_{j}\cdot f\left(X_{t-j},\psi \right)+b \end{array}$$ (1)

where *F*(*X*_*t*_) is the fatigue detection function at time *t*, *X*_*t*_ is the facial feature vector at time *t*, *N* and *M* are the sizes of the considered time windows, *w*_*i*_ and *v*_*j*_ are weight parameters. Functions *g* and *f* are nonlinear functions, Convolutional Neural Networks (CNN), used to extract relevant information from the facial feature vector. *h* is a preprocessing function for handling image features under low brightness and uneven lighting conditions, *θ* and *ψ* are the parameters for functions *g* and *f* respectively, and *b* is a bias term.

**Problem 1**. *The objective is to minimize the prediction error, i.e*.:

$$\begin{array}{c} \text{min}\;\sum_{t=1}^{T}{\left(Y_{t}-{\hat{Y}}_{t}\right)}^{2} \end{array}$$ (2)

*where Y*_*t*_ *is the predicted fatigue state output at time t*, ${\hat{Y}}_{t}$ *is the actual fatigue state at time t, and T is the total observation time*.

### 2.2 Motivation for fatigue dynamics analysis based on posture estimation

- **Limitations of Existing Methods:** Current fatigue monitoring technologies mainly rely on facial feature analysis, such as the degree of eye closure or facial expressions [12–14]. However, these methods often overlook changes in the driver’s overall body posture, such as slumped shoulders or forward-leaning head, which can be signs of fatigue after long periods of driving [15, 16]. These subtle but crucial body language signals are often missed by traditional facial analysis methods, leading to incomplete and inaccurate fatigue monitoring.
- **Technological Innovation:** The research employs advanced posture estimation technology, enhancing not only the identification of facial fatigue signs, such as yawning, but also analyzing overall changes in the driver’s body posture. This approach captures subtle fatigue signals that may appear after prolonged driving, thereby providing a more comprehensive and precise fatigue monitoring. Furthermore, compared to traditional methods, the technology demonstrates superior adaptability and accuracy in complex, dynamic driving environments, marking a significant technological innovation in fatigue detection.

### 2.3 Mathematical implementation of fatigue dynamics analysis based on posture estimation

First, define the posture estimation function *P*(*X*_*t*_) as:

$$\begin{array}{c} P\left(X_{t}\right)=\sum_{i=1}^{N}\alpha_{i}\cdot \text{ReLU}(\sum_{j=1}^{M}W_{ij}*X_{t-j}+b_{i}) \end{array}$$ (3)

where *X*_*t*_ is the image frame at time *t*, *N* and *M* represent the number and size of filters respectively, *W*_*ij*_ and *b*_*i*_ are the weights and biases of the convolution layer, ReLU is the activation function.

Next, define a facial fatigue feature detection function *F*(*X*_*t*_):

$$\begin{array}{cc} F(X_{t}) & =\sigma (\sum_{k=1}^{K}\beta_{k}\cdot \text{Tanh}(\sum_{l=1}^{L}U_{kl}\cdot P\left(X_{t-l}\right)+c_{k}))\\ & =\sigma (\sum_{k=1}^{K}\beta_{k}\cdot \frac{e^{\sum_{l=1}^{L}U_{kl}\cdot P\left(X_{t-l}\right)+c_{k}}-e^{-(\sum_{l=1}^{L}U_{kl}\cdot P\left(X_{t-l}\right)+c_{k})}}{e^{\sum_{l=1}^{L}U_{kl}\cdot P\left(X_{t-l}\right)+c_{k}}+e^{-(\sum_{l=1}^{L}U_{kl}\cdot P\left(X_{t-l}\right)+c_{k})}}) \end{array}$$ (4)

where *σ* is the Sigmoid activation function, used for processing nonlinear features, *K* and *L* are parameters of the network layers, *U*_*kl*_ and *c*_*k*_ are the weights and biases.

Finally, combine body posture and facial fatigue features to assess fatigue level:

$$\begin{array}{cc} Y_{t} & =\gamma \cdot (\sum_{m=1}^{Q}\delta_{m}\cdot F\left(X_{t-m}\right))+\lambda \cdot (\sum_{n=1}^{R}\epsilon_{n}\cdot \text{Softmax}(V_{n}*P\left(X_{t-n}\right)+d_{n}))\\ & =\gamma \cdot (\sum_{m=1}^{Q}\delta_{m}\cdot \sigma (\sum_{k=1}^{K}\beta_{k}\cdot \text{Tanh}(\sum_{l=1}^{L}U_{kl}\cdot P\left(X_{t-m-l}\right)+c_{k})))\\ & \;+\lambda \cdot (\sum_{n=1}^{R}\epsilon_{n}\cdot \frac{e^{V_{n}*P\left(X_{t-n}\right)+d_{n}}}{\sum_{o=1}^{R}e^{V_{o}*P\left(X_{t-o}\right)+d_{o}}}) \end{array}$$ (5)

where *Y*_*t*_ is the fatigue assessment output at time *t*. In this context, *Y*_*t*_ is a scalar representing the fatigue level at time *t*. *γ*, λ, *δ*_*m*_, and *ϵ*_*n*_ are learning parameters, and the Softmax function is used for normalizing the output. *V*_*n*_ and *d*_*n*_ are the weights and biases of another layer of the network.

**Problem 2**. *Given a series of image frames X*_*t*_, *the task is to maximize the accuracy of the fatigue assessment output Y*_*t*_ *while minimizing the prediction error. This can be expressed as the following optimization problem*:

$$\begin{array}{c} \underset{\alpha_{i},\beta_{k},U_{kl},V_{n},\gamma ,\lambda }{\text{max}}\;(\sum_{t=1}^{T}Y_{t})-\underset{\delta_{m},\epsilon_{n},W_{ij},b_{i},c_{k},d_{n}}{\text{min}}\;(\sum_{t=1}^{T}{\left(Y_{t}-{\hat{Y}}_{t}\right)}^{2}) \end{array}$$ (6)

*where Y*_*t*_ *is the scalar fatigue assessment output at time t*, ${\hat{Y}}_{t}$ *is the actual fatigue state at time t, and T is the total observation time*.

In the proposed optimization problem, the following theorems can be introduced to demonstrate that the solution exists, is unique, bounded, and ensures convergence.

The IIAAF model’s mathematical framework, defined in Eqs 3, 4 and 5, incorporates advanced calculus to ensure its applicability and effectiveness in driver fatigue detection. The following theorems articulate these advanced mathematical principles:

**Theorem 1** (Differential Optimization of the Fatigue Detection Model). *In the optimization problem defined by* 6, *the solution set* $\left\{\alpha_{i}^{*},\beta_{k}^{*},U_{kl}^{*},V_{n}^{*},\gamma^{*},\lambda^{*}\right\}$ *maximizes the differential accuracy of the fatigue assessment output Y*_*t*_. *This is expressed as the derivative of Y*_*t*_ *with respect to each parameter, ensuring that the rate of change in fatigue detection accuracy is optimized at every instant*.

$$\begin{array}{c} \frac{dY_{t}}{d\theta }=0,\;for\;\theta \in \left\{\alpha_{i},\beta_{k},U_{kl},V_{n},\gamma ,\lambda \right\} \end{array}$$ (7)

*where dY*_*t*_/*dθ* *represents the partial derivative of the fatigue assessment output with respect to the model parameters*.

**Theorem 2** (Convergence Analysis through Integral Calculus). *The boundedness and convergence of the IIAAF model, as defined in* 6, *can be analyzed through integral calculus. The cumulative effect of incremental changes in model parameters on the fatigue assessment accuracy is bounded, ensuring the model’s stability over continuous operational periods*.

$$\begin{array}{c} \int_{\alpha_{i}}^{\alpha_{i}^{*}}\int_{\beta_{k}}^{\beta_{k}^{*}}\cdots \int_{\lambda }^{\lambda^{*}}\frac{\partial Y_{t}}{\partial \theta }d\theta \;is\;finite\;and\;bounded \end{array}$$ (8)

*where* $\frac{\partial Y_{t}}{\partial \theta }$ *represents the partial derivative of Y*_*t*_ *with respect to each parameter θ*.

### 2.4 Illumination Intelligent Adaptation and Analysis Framework (IIAAF)

#### 2.4.1 Motivation for per-pixel dynamic illumination adjustment technology

- **Limitations of Existing Methods:** Traditional illumination adjustment techniques usually focus on overall brightness adjustment or only target key facial areas for localized adjustment [17, 18]. These methods often fail to adapt effectively in complex nighttime driving environments, especially when encountering sudden changes in lighting (e.g., flickering streetlights or headlights from oncoming vehicles), thus impacting the accuracy of driver facial feature recognition.
- **Technological Innovation:** The proposed per-pixel dynamic illumination adjustment technology employs advanced image processing algorithms, capable of meticulously adjusting the brightness and contrast of each pixel and responding in real-time to rapid changes in lighting conditions [19]. This technology ensures clear visibility of the driver’s facial features while adapting to the fluctuating lighting in complex nighttime driving environments [20]. The method represents a significant technological breakthrough in intelligent driving assistance systems, particularly in fatigue monitoring and facial recognition.

#### 2.4.2 Mathematical implementation of per-pixel dynamic illumination adjustment technology

To enhance the accuracy of driver facial feature recognition in nighttime driving environments, the research introduces an advanced per-pixel dynamic illumination adjustment technology. This technique goes beyond adjusting the brightness of key facial areas and meticulously adjusts the brightness and contrast for every pixel captured by the camera. This process involves complex image processing and real-time brightness adjustment algorithms to cope with rapidly changing lighting conditions.

First, define the image brightness adjustment function *L*(*I*_*xy*_, *t*):

$$\begin{array}{c} L\left(I_{xy},t\right)=\sum_{p=1}^{P}\theta_{p}\cdot (\text{ReLU}(\sum_{q=1}^{Q}\eta_{pq}*I_{xy,t-q}+\xi_{p})) \end{array}$$ (9)

where *I*_*xy*_ is the brightness value at pixel (*x*, *y*), *t* represents time, *P* and *Q* respectively represent the depth and size of convolution layers, *η*_*pq*_ and *ξ*_*p*_ are the weights and biases of the convolution layer. This way, the system can analyze brightness changes for each pixel.

Next, define a real-time brightness adjustment optimization model:

$$\begin{array}{cc} A(I_{xy},t) & =\sum_{r=1}^{R}\gamma_{r}\cdot (\text{Sigmoid}(\sum_{s=1}^{S}\omega_{rs}\cdot L\left(I_{xy,t-s}\right)+\zeta_{r}))\\ & \;+\sum_{u=1}^{U}\lambda_{u}\cdot (\text{Tanh}(\sum_{v=1}^{V}\mu_{uv}\cdot I_{xy,t-v}+\rho_{u})) \end{array}$$ (10)

This model uses Sigmoid and Tanh activation functions to adjust the brightness and contrast of each pixel in real-time in response to rapid changes in lighting conditions. Here, *R*, *S*, *U*, and *V* are parameters of network layers, *ω*_*rs*_, *μ*_*uv*_, *ζ*_*r*_, and *ρ*_*u*_ are the weights and biases.

**Problem 3**. *Given a series of image frames I*_*xy*,*t*_, *the goal is to maximize the overall brightness and contrast adjustment effect across the entire facial image. This can be expressed as the following optimization problem*:

$$\begin{array}{c} \underset{\theta_{p},\eta_{pq},\xi_{p},\gamma_{r},\omega_{rs},\zeta_{r},\lambda_{u},\mu_{uv},\rho_{u}}{\text{max}}\;(\sum_{t=1}^{T}\sum_{x,y}A\left(I_{xy,t}\right)) \end{array}$$ (11)

*where T is the total observation time, x, y represent pixel coordinates, and A*(*I*_*xy*,*t*_) *is a scalar function that adjusts the brightness and contrast for each pixel at time t. The output of A*(*I*_*xy*,*t*_) *is typically a binary value indicating whether the adjustment criteria are met for pixel* (*x*, *y*) *at time t*.

**Theorem 3** (Optimized Dynamic Illumination Adjustment). *For the real-time brightness adjustment formula* 10, *an optimal parameter set* {*θ*_*p*_, *η*_*pq*_, *ξ*_*p*_, *γ*_*r*_, *ω*_*rs*_, *ζ*_*r*_, λ_*u*_, *μ*_*uv*_, *ρ*_*u*_} *exists, which maximizes the overall illumination adjustment of each pixel in image frames* *I*_*xy*,*t*_. *The optimization condition is represented by the following equation*:

$$\begin{array}{c} \frac{\partial }{\partial \theta }(\sum_{t=1}^{T}\sum_{x,y}A\left(I_{xy,t}\right))=0,\;\forall \theta \in \left\{\theta_{p},\eta_{pq},\xi_{p},\gamma_{r},\omega_{rs},\zeta_{r},\lambda_{u},\mu_{uv},\rho_{u}\right\} \end{array}$$ (12)

*where* $\frac{\partial }{\partial \theta }$ *indicates the partial derivative with respect to each parameter θ, ensuring optimal brightness and contrast adjustment for enhanced visibility and image quality*.

**Corollary 1** (Real-Time Adjustment and Environmental Adaptability). *In the optimization problem of* Eq 11, *the per-pixel dynamic illumination adjustment technology, through implementing the optimization model of* 10, *demonstrates high adaptability to rapidly changing lighting conditions. Specifically, this technology can adapt to environmental lighting fluctuations* Δ*L*_*t*_ *by adjusting the function A*(*I*_*xy*_, *t*), *expressed as*:

$$\begin{array}{c} A\left(I_{xy},t+\Delta t\right)=A\left(I_{xy},t\right)+\frac{\partial A}{\partial L_{t}}\Delta L_{t} \end{array}$$ (13)

*where* Δ*L*_*t*_ *represents changes in lighting, such as flickering streetlights or headlights from oncoming vehicles. This adaptive optimization enhances the accuracy and reliability of driver facial fatigue detection*.

#### 2.4.3 Motivation for the light variation feature learning system

- **Limitations of Existing Methods:** Traditional methods of handling light variation mostly rely on regular adjustments, lacking a deep understanding and adaptability to environmental changes [21]. In nighttime driving environments, especially when faced with sudden changes like flickering streetlights or headlights from oncoming vehicles, these methods often fail to provide stable and accurate image adjustments, affecting the precise identification of driver fatigue states [22, 23].
- **Technological Innovation:** The light variation feature learning system, by combining CNN and time-series analysis models, learns and predicts light variation patterns from historical image data. This approach not only recognizes the current lighting conditions but also predicts potential future light fluctuations, adjusting the image capturing strategy accordingly. This learning and prediction mechanism enables the system to adapt more intelligently and accurately to the continuously changing lighting conditions of nighttime driving, significantly enhancing the stability and accuracy of fatigue detection.

#### 2.4.4 Mathematical implementation of the illumination change feature learning system (Illumination change feature learning system)

The system utilizes CNN and time-series analysis models to deeply learn from historical image data, identifying patterns of light variation in nighttime driving environments to guide real-time image adjustment strategies.

First, define the light variation feature extraction function *G*(*I*_*xy*_, *t*):

$$\begin{array}{c} G\left(I_{xy},t\right)=\sum_{i=1}^{I}\phi_{i}\cdot \text{ReLU}(\sum_{j=1}^{J}\psi_{ij}*I_{xy,t-j}+\kappa_{i}) \end{array}$$ (14)

where *I*_*xy*_ represents the brightness value at pixel (*x*, *y*), *t* is time, *I* and *J* represent the depth and size of convolution layers, *ψ*_*ij*_ and *κ*_*i*_ are the weights and biases.

Further, construct a time-series analysis model for light variation:

$$\begin{array}{c} T\left(I_{xy},t\right)=\sum_{k=1}^{K}\alpha_{k}\cdot \text{Softmax}(\sum_{l=1}^{L}\beta_{kl}\cdot G\left(I_{xy,t-l}\right)+\lambda_{k}) \end{array}$$ (15)

where *α*_*k*_, *β*_*kl*_, and λ_*k*_ are parameters of the time-series analysis model, *K* and *L* are parameters of network layers.

Next, the research combines feature extraction and time-series analysis to define a comprehensive light variation prediction model:

$$\begin{array}{c} P\left(I_{xy},t\right)=\sum_{m=1}^{M}\gamma_{m}\cdot \text{Tanh}(\sum_{n=1}^{N}\delta_{mn}\cdot T\left(I_{xy,t-n}\right)+\epsilon_{m}) \end{array}$$ (16)

This model uses Tanh activation function to enhance prediction accuracy, where *γ*_*m*_, *δ*_*mn*_, and *ϵ*_*m*_ are weights and biases, *M* and *N* are parameters of network layers.

Lastly, define the light variation pattern learning optimization model:

$$\begin{array}{c} L\left(I_{xy},t\right)=\sum_{r=1}^{R}\mu_{r}\cdot (\text{Sigmoid}(\sum_{s=1}^{S}\nu_{rs}\cdot P\left(I_{xy,t-s}\right)+\xi_{r})) \end{array}$$ (17)

where *μ*_*r*_, *ν*_*rs*_, and *ξ*_*r*_ are parameters of the pattern learning model, *R* and *S* are parameters of network layers.

**Problem 4**. *Given a series of image frames I*_*xy*,*t*_, *the objective is to maximize the predictive power and accuracy of the light variation pattern learning model L*(*I*_*xy*,*t*_). *This can be expressed as the following optimization problem*:

$$\begin{array}{c} \underset{\phi_{i},\psi_{ij},\kappa_{i},\alpha_{k},\beta_{kl},\lambda_{k},\gamma_{m},\delta_{mn},\epsilon_{m},\mu_{r},\nu_{rs},\xi_{r}}{\text{max}}\;(\sum_{t=1}^{T}\sum_{x,y}L\left(I_{xy,t}\right)) \end{array}$$ (18)

*where T is the total observation time*, *x*, *y represent pixel coordinates, and L*(*I*_*xy*,*t*_) *is a scalar function that represents the model’s prediction accuracy or power for each pixel at time* *t*. *The value of L*(*I*_*xy*,*t*_) *can vary depending on the model’s confidence or other criteria*.

**Corollary 2** (Environmental Light Adaptability). *In optimization formulation* 18, *the light variation feature learning system exhibits significant adaptability to environmental light changes. This adaptability is not only limited to recognizing current lighting conditions but can also predict and adapt to future light changes. Specifically, through the model L*(*I*_*xy*_, *t*), *the system can predict light variations at a future time t* + Δ*t*, *expressed as*:

$$\begin{array}{c} L\left(I_{xy},t+\Delta t\right)\approx L\left(I_{xy},t\right)+\frac{\partial L}{\partial t}\Delta t \end{array}$$ (19)

*where* $\frac{\partial L}{\partial t}$ *represents the time derivative, reflecting the dynamic nature of light changes*.

**Corollary 3** (Prediction Accuracy and Stability). *Based on the comprehensive prediction model P*(*I*_*xy*_, *t*) *of the light variation feature learning system, the system not only accurately predicts short-term light changes but also exhibits excellent long-term stability. This effectiveness is attributed to the model’s deep network structure and comprehensive time-series analysis, thereby ensuring stability and accuracy in fatigue detection in complex nighttime driving environments*.

## 3 Algorithm pseudocode and complexity

**Fig 2:**
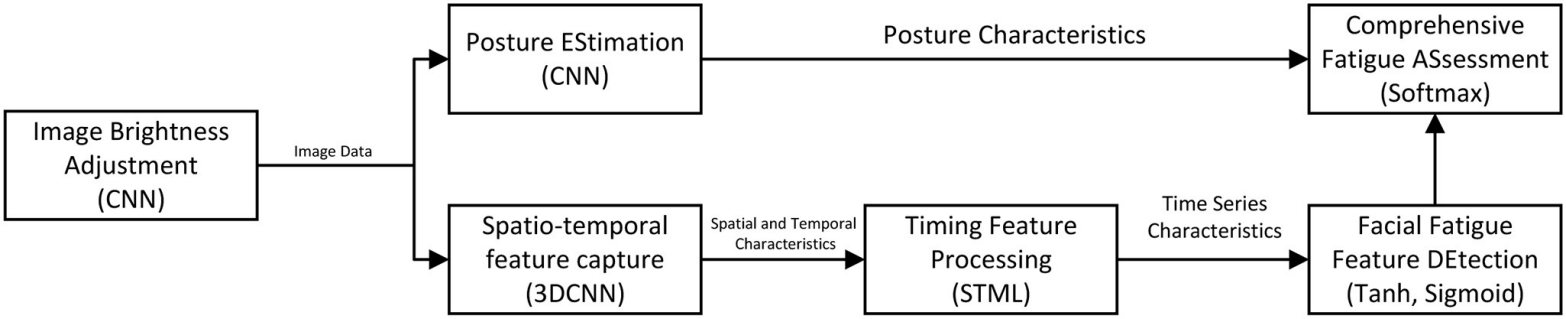
Neural network architecture for driver fatigue detection.

The specific process and methods for constructing the experiment are shown in Fig 2.

The time complexity of Algorithm 1 primarily depends on its constituent functions. The time complexity of each call to the PoseEstimation and FeatureDetection functions is *O*(*NM*) and *O*(*KL*), respectively, where *N*, *M*, *K*, *L* are network layer parameters. Since the FatigueDetection function calls both of these functions, its time complexity is *O*(*NM* + *KL*). The Main function calls FatigueDetection *T* times, so the total time complexity is *O*(*T*(*NM* + *KL*)). The space complexity is determined by the parameters used in the algorithm and the outputs stored. The algorithm stores parameters *α*_*i*_, *β*_*k*_, *U*_*kl*_, *V*_*n*_, *γ*, λ, whose space complexity is *O*(*N* + *M* + *K* + *L*). It also stores the output *Y*_*t*_ for each frame, a total of *T* frames, so the space complexity is *O*(*T*). The overall space complexity is *O*(*N* + *M* + *K* + *L* + *T*).

**Algorithm 1:** Pose Estimation and Facial Fatigue State Detection Algorithm

 **Data**: A sequence of continuous video frames {*X*_1_, *X*_2_, …, *X*_*T*_}

 **Result**: Predicted fatigue state outputs {*Y*_1_, *Y*_2_, …, *Y*_*T*_}

**1** **Function** Main():

**2**  Initialize parameters *α*_*i*_, *β*_*k*_, *U*_*kl*_, *V*_*n*_, *γ*, λ

**3**  **for** *t* = 1 **to** *T* **do**

**4**   *Y*_*t*_ ← FatigueDetection(*X*_*t*_)

**5**  **end**

**6**  Minimize optimization problem (6)

**7** **Function** FatigueDetection(*X*_*t*_):

**8**  *P*_*t*_ ← PoseEstimation(*X*_*t*_)

**9**  *F*_*t*_ ← FeatureDetection(*P*_*t*_)

**10**  **return** using (5) to calculate *Y*_*t*_

**11** **Function** PoseEstimation(*X*_*t*_):

  // Pose estimation function

**12**  **return** using (3) to calculate *P*(*X*_*t*_)

**13** **Function** FeatureDetection(*P*_*t*_):

  // Facial fatigue feature detection

**14**  **return** using (1) and (4) to calculate *F*(*X*_*t*_)

Algorithm 2 consists of two main parts: Per-Pixel Dynamic Light Adjustment (LightAdjustment) and Light Variation Feature Learning System (FeatureLearning). The time complexity of LightAdjustment is *O*(*PQ* + *RS* + *UV*), while that of FeatureLearning is *O*(*IJ* + *KL* + *MN*). The Main function calls these two functions *T* times, so the total time complexity is *O*(*T*(*PQ* + *RS* + *UV* + *IJ* + *KL* + *MN*)). The space complexity is determined by the parameters used in the algorithm and the stored image frames. The algorithm stores parameters for both parts, including *θ*_*p*_, *η*_*pq*_, *ξ*_*p*_, *γ*_*r*_, *ω*_*rs*_, *ζ*_*r*_, λ_*u*_, *μ*_*uv*_, *ρ*_*u*_ and *ϕ*_*i*_, *ψ*_*ij*_, *κ*_*i*_, *α*_*k*_, *β*_*kl*_, λ_*k*_, *γ*_*m*_, *δ*_*mn*_, *ϵ*_*m*_, *μ*_*r*_, *ν*_*rs*_, *ξ*_*r*_, with a space complexity of *O*(*P* + *Q* + *R* + *S* + *U* + *V* + *I* + *J* + *K* + *L* + *M* + *N*). It also stores the adjusted image frames $\left\{I_{1}^{\prime },I_{2}^{\prime },\ldots ,I_{T}^{\prime }\right\}$, so the total space complexity is *O*(*T* + *P* + *Q* + *R* + *S* + *U* + *V* + *I* + *J* + *K* + *L* + *M* + *N*). The algorithms trade space for time efficiency.

**Algorithm 2**: Per-Pixel Light Adjustment and Light Variation Feature Learning Algorithm

 **Data**: Predicted fatigue state outputs from previous Algorithm 1 {*Y*_1_, *Y*_2_, …, *Y*_*T*_}, A sequence of continuous video frames {*I*_1_, *I*_2_, …, *I*_*T*_}

 **Result**: Image frames after light adjustment and feature learning $\left\{I_{1}^{\prime },I_{2}^{\prime },\ldots ,I_{T}^{\prime }\right\}$

**1 Function** Main():

  // Per-pixel dynamic light adjustment part

**2**  **for** *t* = 1 **to** *T* **do**

**3**   $I_{t}^{\prime }$ ← LightAdjustment(*I*_*t*_)

**4**  **end**

  // Light variation feature learning system part

**5**  **for** *t* = 1 **to** *T* **do**

**6**   FeatureLearning($I_{t}^{\prime }$)

**7**  **end**

**8** **Function** *Part A* LightAdjustment(*I*_*t*_):

  // Per-pixel brightness adjustment

**9**  Using (9), (10) and (11)

**10**  **return** adjusted image frame $I_{t}^{\prime }$

**11** **Function** *Part B* FeatureLearning($I_{t}^{\prime }$):

  // Light variation feature learning

**12**  Using (14), (15) and (16)

**13**  Optimize parameters in (17)

**14**  Update light variation pattern learning model *L*(*I*_*xy*_, *t*)

**15**  Apply optimization strategy in (18)

## 4 Experimental results

### 4.1 Datasets

#### 4.1.1 Yawn dataset

The Yawn Dataset, available on the Kaggle official website, includes two categories: Yawn and no-Yawn. David Hiram Vazquez Santana. (2021. Oct). Yawn Dataset, V1.0. from https://www.kaggle.com/datasets/davidvazquezcic/yawn-dataset/data. The Yawn category contains 2528 JPG format images, and the no-Yawn category contains 2591 JPG format images. Each category features diverse racial, gender, and age representations in “mouth” characteristics. This dataset is solely used for testing the classification effectiveness of the model proposed by Algorithm 1.

#### 4.1.2 YawDDR dataset

The YawDDR dataset (Shabnam Abtahi, Mona Omidyeganeh, Shervin Shirmohammadi, Behnoosh Hariri, August 1, 2020, “YawDD: Yawning Detection Dataset”, IEEE Dataport, doi: https://dx.doi.org/10.21227/e1qm-hb90.) has been developed as an extension of the standard YawDD dataset, a publicly available dataset for yawning detection. The YawDDR dataset serves the purpose of validating algorithms related to face detection, facial feature extraction, and yawning detection. It consists of action videos captured from volunteers of diverse genders, ages, nationalities, and ethnicities, totaling 351 video clips. The dataset records individuals in stationary vehicles under daylight conditions with slight variations in illumination. Each driver is recorded in three or four videos, featuring different mouth actions such as talking, yawning, and yawning while talking. Keyframes are extracted and limb and facial features are highlighted according to experimental requirements. To simulate the environment proposed in the article, the dataset undergoes corresponding processing, with specific methods given in the experimental section.

### 4.2 Experimental results and analysis

To validate the method proposed in this paper, three experiments were designed in the environment as shown in Table 1. The first experiment tests the fatigue dynamics analysis method based on posture estimation, training the model proposed by Algorithm 1, and presents the training results. The second experiment tests the dynamic light adjustment method for highlighting facial features, adding Algorithm 2-a on the basis of Experiment 1, and observing the model detection results. The third experiment tests the dynamic light adjustment method for highlighting facial features, adding Algorithm 2-b on the basis of Experiment 1, to see if the addition of Algorithm 2-b improves detection results.

**Table 1:** Experimental parameter settings.

Parameter	Value
Dataset size	500 video clips
Video resolution	1280x720 pixels
Average duration per video	15 minutes
Time window size *N*	10 seconds
Frame extraction rate	10 frames/second
Data augmentation	Random cropping, color adjustment
Proportion of positive samples	50%
Proportion of negative samples	50%
CNN layers (light adjustment part)	5 layers
CNN layers (feature learning part)	6 layers
Number of filters per layer (light adjustment part)	32
Filter size (light adjustment part)	3x3
Stride (light adjustment part)	1
Padding (light adjustment part)	Same
Number of filters per layer (feature learning part)	64, 128, 256, 512, 512, 512
Filter size (feature learning part)	3x3
Stride (feature learning part)	1
Padding (feature learning part)	Same
Activation function	ReLU
Feature extraction layer parameters *Φ* (feature learning part)	512 neurons
Fusion layer parameters *Ψ* (feature learning part)	256 neurons
Training epochs	110 epochs
Batch size	16
Learning rate	0.0005
Optimizer	Adam
Loss function	Mean squared error
Dropout rate	0.4
Validation set proportion	15%
Early stopping threshold	15 epochs
Regularization method	L2 regularization
L2 regularization rate	0.02
Data augmentation techniques	Random rotation, color enhancement
Rotation range	±10 degrees
Color enhancement range	0.9–1.1
Input data format	Color images
Number of image channels	3 (RGB)
Keyframe extraction strategy	Based on motion detection
Evaluation metrics	Accuracy, F1 score
Test set proportion	15%
Data preprocessing	Standardization, denoising
Denoising method	Bilateral filtering
Standardization range	-1 to 1
Experimental environment	NVIDIA GeForce RTX 3080

#### 4.2.1 Testing the fatigue dynamics analysis method based on posture estimation

**Fig 3:**
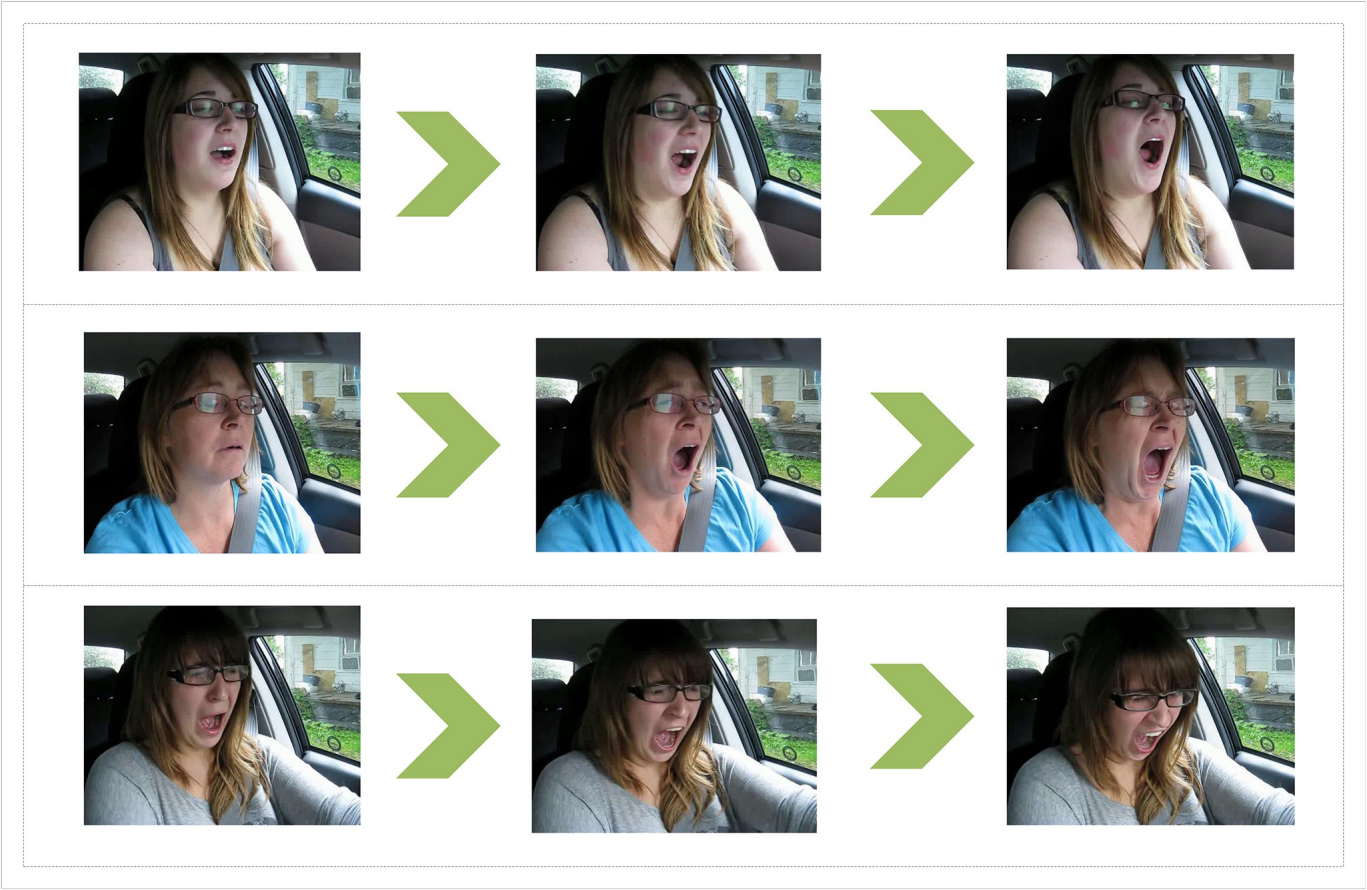
Sample images from Experiment 2 dataset (keyframes at different moments, image source: Yawning detection dataset (DOI: https://dx.doi.org/10.21227/e1qm-hb90), licensed under CC BY 4.0.).

In this experiment, the Yawn dataset remains unchanged, while for the YawDDR dataset, keyframes are extracted focusing on the expressions and body posture of the subjects. Keyframe extraction results from the YawDDR dataset are shown in Fig 3, displaying keyframes from three different video segments.

**Fig 4:**
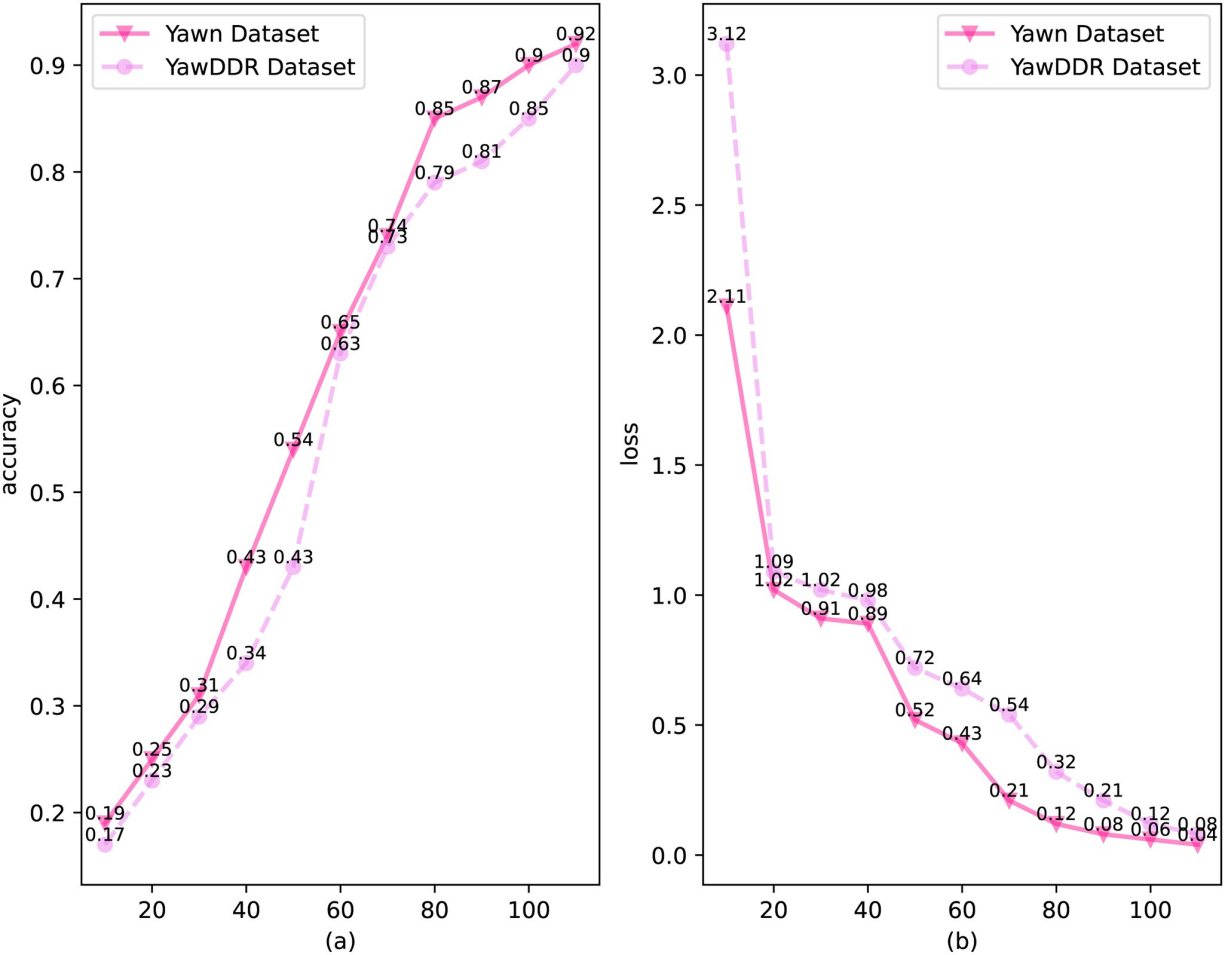
Training results of Algorithm 1 (Yawn dataset: Model detection results on the Yawn dataset; YawDDR dataset: Model detection results on the YawDDR dataset).

After preparing the dataset for the simulated environment, it is necessary to establish and train the model structure proposed by Algorithm 1. The training results of the model are shown in Fig 4(b), where the loss value on the test set is below 0.1, indicating good performance of the model. Observing Fig 4(a), the model’s accuracy on the Yawn dataset is higher than that on the YawDDR dataset, indicating that the model performs better on the Yawn dataset than on the YawDDR dataset.

#### 4.2.2 Dynamic light adjustment for facial highlighting test

**Fig 5:**
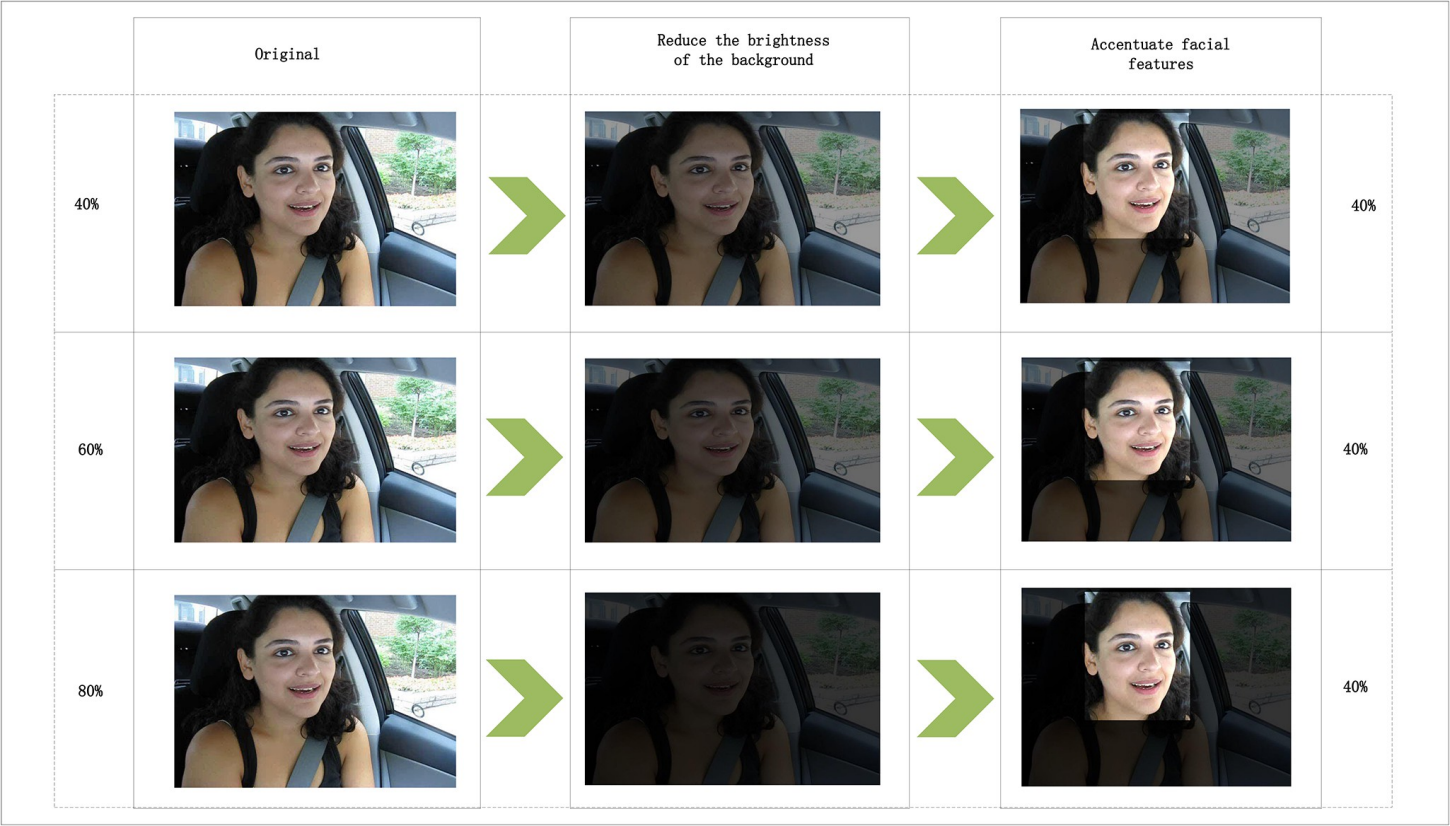
Sample images from Experiment 2 dataset (left column indicates the degree of brightness reduction, right column indicates the degree of brightness increase, Image source: Yawning Detection Dataset (DOI: https://dx.doi.org/10.21227/e1qm-hb90), licensed under CC BY 4.0.)).

To verify the performance of Algorithm 2, the research further processed the keyframes of the YawDDR dataset, first detecting facial features, then adjusting the background color of the keyframes, and finally increasing the brightness of the facial feature areas. Dataset processing examples are shown in Fig 5, where the numbers on the left indicate a 40%, 60%, 80% reduction in background brightness, and the numbers on the right indicate a 1.4x, 1.6x, 1.8x increase in facial brightness.

**Fig 6:**
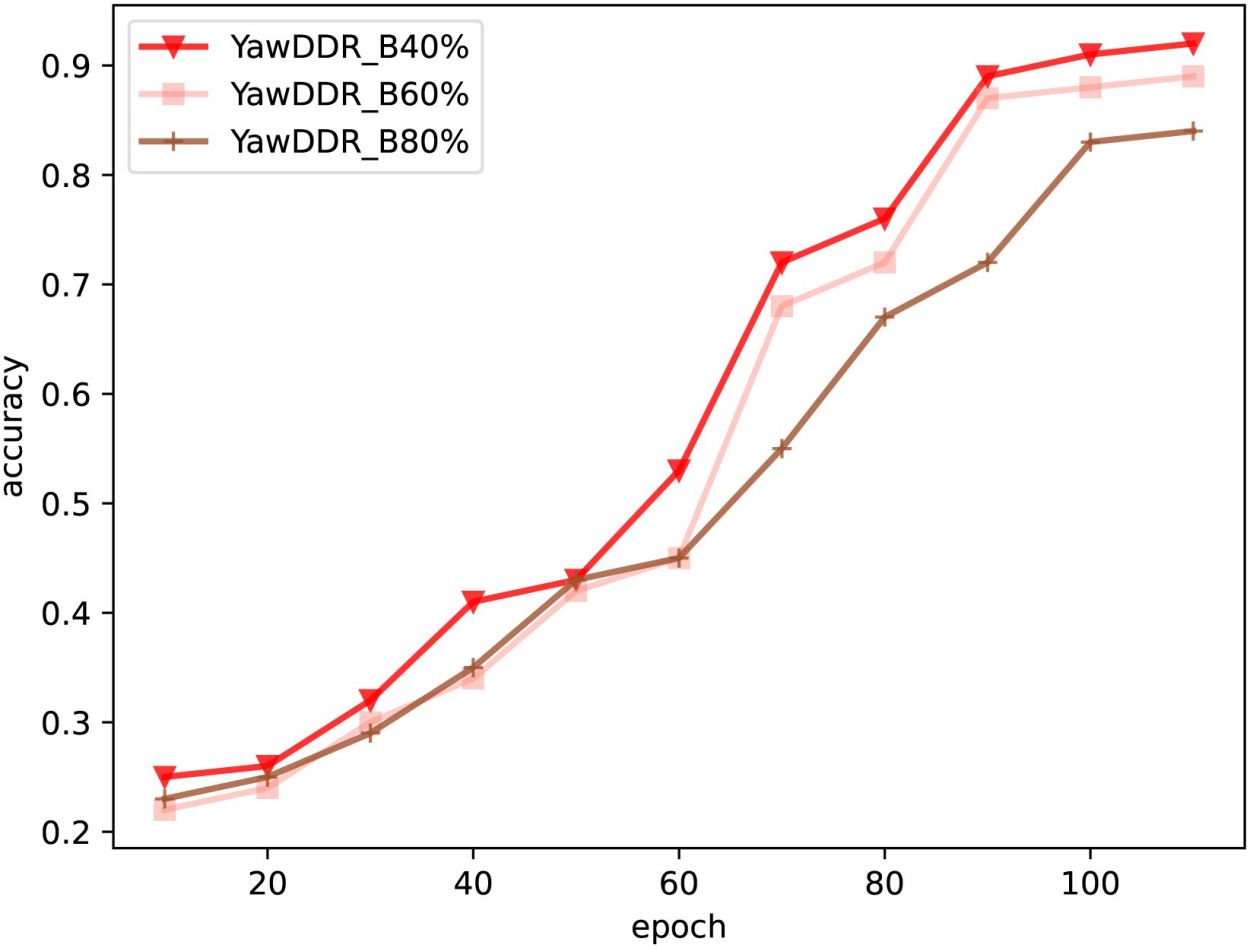
Detection results of Experiment 2 (Yawn_B40%: Background brightness reduced by 40%, facial brightness increased by 40%. Yawn_B60%: Background brightness reduced by 60%, facial brightness increased by 60%. Yawn_B80%: Background brightness reduced by 80%, facial brightness increased by 80%).

After processing the dataset as described above, the model was trained, and the training results are shown in Fig 6.

#### 4.2.3 Light variation adjustment system test

**Fig 7:**
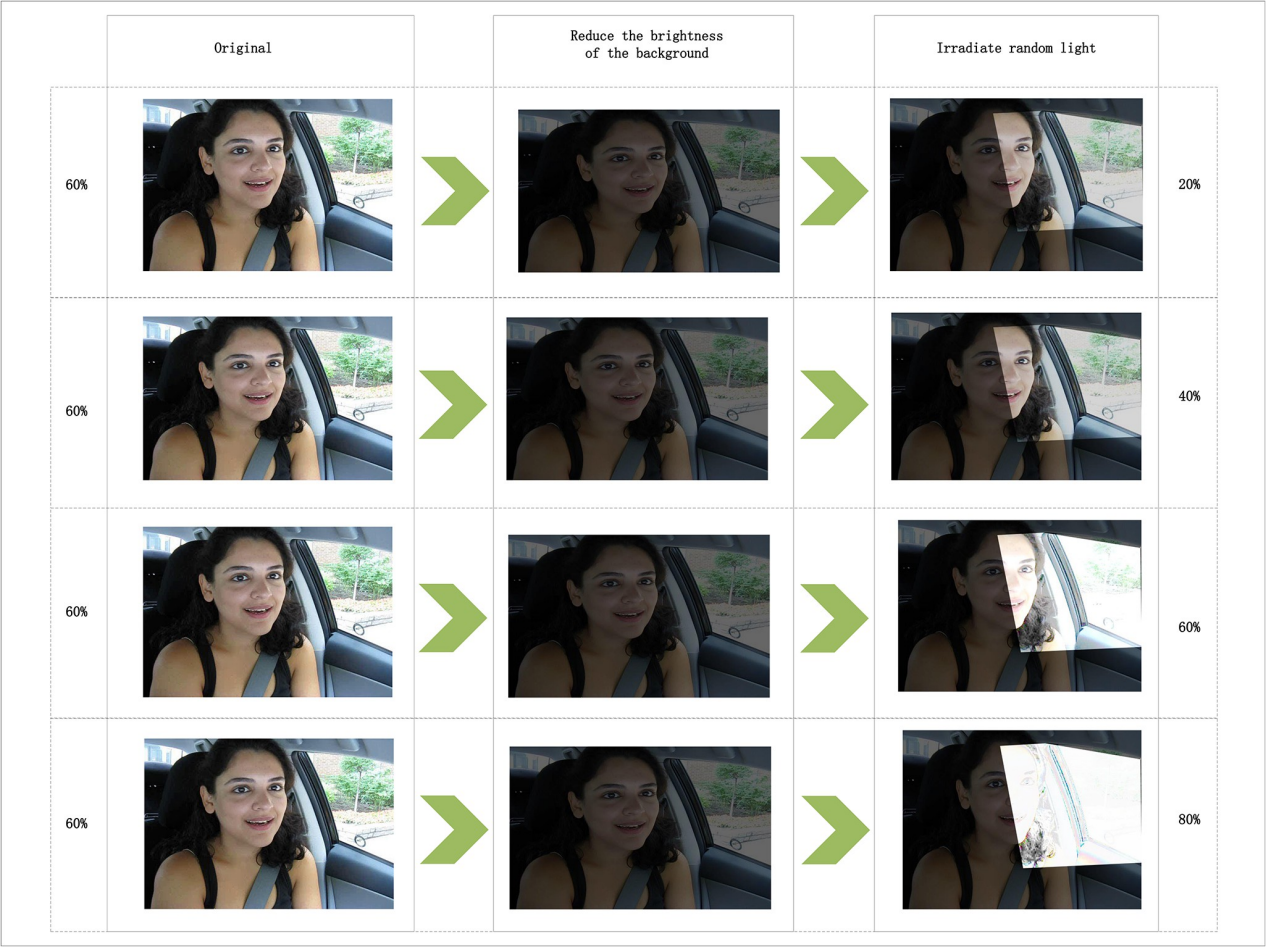
Sample images from Experiment 3 dataset (left column indicates the degree of brightness reduction, right column indicates the degree of brightness increase, Image source: Yawning Detection Dataset (DOI: https://dx.doi.org/10.21227/e1qm-hb90), licensed under CC BY 4.0.)).

To verify the performance of Algorithm 3, based on the dataset processing method of Experiment 2, the research adjusted the head lighting, as shown in Fig 7. The left and right columns of the figure are similar to those in Experiment 2, with the background brightness reduced by 60%, and facial brightness adjusted to 1.2x, 1.4x, 1.6x, 1.8x.

**Fig 8:**
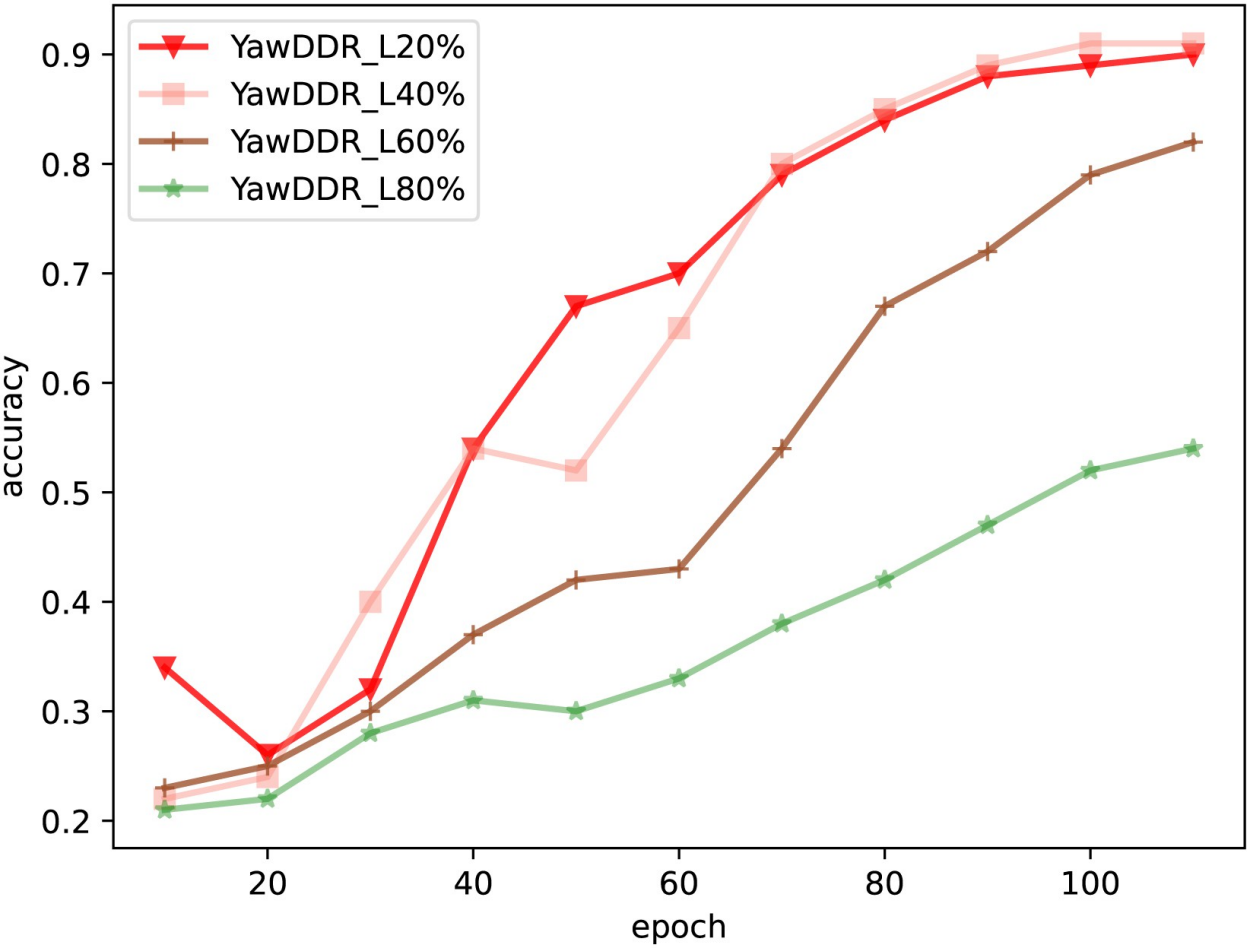
Detection results of Experiment 3 (YawnDDR_L20%: Background brightness reduced by 60%, facial brightness adjusted to 1.2x. YawnDDR_L40%: Background brightness reduced by 60%, facial brightness adjusted to 1.4x. YawnDDR_L60%: Background brightness reduced by 60%, facial brightness adjusted to 1.6x. YawnDDR_L80%: Background brightness reduced by 60%, facial brightness adjusted to 1.8x.).

After processing the dataset as mentioned, the model was retrained, and the training results are shown in Fig 8. YawnDDR_L20% indicates that the background brightness was reduced by 60%, and facial brightness was adjusted to 1.2x. YawnDDR_L40% indicates a background reduction of 60% and facial brightness adjustment of 1.4x. YawnDDR_L60% and YawnDDR_L80% follow a similar pattern. Observing the figure, it is evident that the best model results are achieved with 1.4x light brightness when the background is reduced below 60%. With a light brightness of 1.2x, the model detection results were slightly poorer due to lower light intensity. When the brightness is too high, the learning effect of the model is poor, and the detection accuracy decreases significantly.

**Table 2:** Comparison of method’s accuracy.

Method	Accuracy	Loss Value	F1 Value	MSE Value
Our Method	95.1%	0.05	0.95	0.05
Our Method without Algorithm 2-B	93.3%	0.06	0.93	0.06
Our Method without Algorithm 2-A	92.2%	0.07	0.92	0.07
Our Method without Algorithm 2-A and Algorithm 2-B	92.1%	0.07	0.92	0.07
Akrout and Mahdi (2016) [24]	83.0%	N/A	N/A	N/A
Yang et al. (2020) [25]	83.4%	N/A	N/A	N/A
Zhang and Su (2017) [26]	88.6%	N/A	N/A	N/A
Anber et al.(2022) [27]	91.0%	N/A	N/A	N/A
Kielty et al. (2023) [28]	92.0%	N/A	N/A	N/A
Majeed et al.(2023) [29]	95.0%	N/A	N/A	N/A

Finally, the research summarized the comparison results of the detection accuracy between the method proposed in this paper and other methods, as shown in Table 2. All experiments were conducted using the YawDDR dataset, which is specifically designed for detecting and analyzing driver fatigue states. From the table, it is evident that Our Method outperforms the methods cited in [24–28]. However, the variants of Our Method without Algorithm 2-A, Algorithm 2-B, or both, did not surpass the performance of [29]. It is noteworthy that while Our Method exclusively employs deep learning models for fatigue driving detection, other studies, such as [30], though not included in the table, combine deep learning feature extraction with machine learning classification algorithms. Although this approach may achieve high accuracy, it lacks the end-to-end classification capability, presenting limitations in real-world applications. In a normal computing environment, the computation time on the research’s experimental platform is approximately 0.5 seconds. This ensures that drivers can receive timely feedback on their fatigue status, addressing a crucial aspect of real-time monitoring.

### 4.3 Discussion

- **Advantages of IIAAF:** As highlighted in the experimental section, the application of IIAAF extends beyond mere dataset image analysis. Crucially, the approach involves extracting keyframes from continuous video streams, closely mimicking real-world driving scenarios. This method ensures the dynamic and real-time processing of visual data, mirroring the constantly changing conditions of night driving.
- **Practical Implementation of IIAAF:** The practical implementation of IIAAF in real-world scenarios is notably straightforward, particularly its integration within existing in-vehicle assistance systems. This ease of implementation is a significant advantage, as it allows for the seamless adoption of the IIAAF framework in modern vehicles without the need for extensive modifications or specialized equipment. The framework’s adaptability to different vehicle models and its compatibility with standard automotive cameras further enhance its practical utility.
- **Limitations of the IIAAF:** Despite its advantages, IIAAF has certain limitations. 1) Firstly, the framework’s performance is heavily reliant on the quality of the input video data. In scenarios where the video quality is severely compromised, such as extremely low light or high glare conditions, the accuracy of fatigue detection may be affected. 2) Secondly, the real-time processing capabilities of IIAAF, though advanced, are subject to the computational power available within the vehicle’s assistance system. In vehicles with less advanced computational resources, the response time of the IIAAF system might be slower, potentially impacting its effectiveness in promptly alerting drivers about their fatigue levels. 3)Lastly, the current version of IIAAF primarily focuses on visual indicators of fatigue. While effective, this approach may not capture all aspects of driver fatigue, which can also manifest in non-visual forms such as cognitive or emotional fatigue. Future enhancements of the system could integrate additional sensors or data sources to address this aspect more comprehensively.
  These limitations are inherent to the current state of technology and the complex nature of fatigue detection, rather than a reflection of the framework’s design or implementation.

## 5 Conclusion

This study focuses on the challenges of monitoring driver fatigue during nighttime driving, especially under low-light conditions. By introducing the “Illumination Intelligent Adaptation and Analysis Framework (IIAAF)”, the research implements fatigue dynamics analysis based on posture estimation, per-pixel dynamic light adjustment techniques, and a light variation feature learning system combining CNN and time-series analysis. Experimental results demonstrate that these methods surpass existing technologies in enhancing the accuracy and practicality of nighttime driving fatigue monitoring, significantly contributing to the development of intelligent driving assistance systems and the improvement of nighttime driving safety. Future research directions will consider conducting supplementary experiments in real driving scenarios, combining them with the methods proposed in this paper. This will further enhance the completeness of the framework.

## Supporting information

S1 Appendix Mathematical proofs. (PDF)

## References

[1] Mello M. T., Narciso F. V., Tufik S., Paiva T., Spence D. W., BaHammam A. S., et al. (2013). Sleep disorders as a cause of motor vehicle collisions. *International Journal of Preventive Medicine*, 4(3), 246. 23626880 PMC3634162

[2] Zhang, Q., Wang, Y., Wu, C., & Zhang, H. (2019). Research on Maximum Driving Time Based on Driving Fatigue Model from Field Experiment. In *2019 5th International Conference on Transportation Information and Safety (ICTIS)* (pp. 1068–1073). IEEE.

[3] Rezaee, K., Khosravi, M. R., Attar, H., & Almatarneh, S. (2022). EEG-Based Driving Fatigue Recognition Using Hybrid Deep Transfer Learning Approach. In *2022 International Engineering Conference on Electrical*, *Energy*, *and Artificial Intelligence (EICEEAI)* (pp. 1–6). IEEE.

[4] You F., Li Y.-h., Huang L., Chen K., Zhang R.-h., & Xu J.-m. (2017). Monitoring drivers’ sleepy status at night based on machine vision. *Multimedia Tools and Applications*, 76, 14869–14886. doi: 10.1007/s11042-016-4103-x

[5] Ma, X., Chau, L.-P., & Yap, K.-H. (2017). Depth video-based two-stream convolutional neural networks for driver fatigue detection. In *2017 International Conference on Orange Technologies (ICOT)* (pp. 155–158). IEEE.

[6] Sikander G., & Anwar S. (2018). Driver fatigue detection systems: A review. *IEEE Transactions on Intelligent Transportation Systems*, 20(6), 2339–2352. doi: 10.1109/TITS.2018.2868499

[7] Matthews, G., Wohleber, R., Lin, J., Funke, G., & Neubauer, C. (2019). Monitoring task fatigue in contemporary and future vehicles: a review. In G. Stanton (Ed.), *Advances in Human Factors in Simulation and Modeling: Proceedings of the AHFE 2018 International Conferences on Human Factors and Simulation and Digital Human Modeling and Applied Optimization*, *Held on July 21–25*, *2018*, *in Loews Sapphire Falls Resort at Universal Studios*, *Orlando*, *Florida*, *USA* (pp. 101–112). Springer.

[8] Doudou M., Bouabdallah A., & Berge-Cherfaoui V. (2020). Driver drowsiness measurement technologies: Current research, market solutions, and challenges. *International Journal of Intelligent Transportation Systems Research*, 18, 297–319. doi: 10.1007/s13177-019-00199-w

[9] Karuppusamy N. S., & Kang B.-Y. (2020). Multimodal system to detect driver fatigue using EEG, gyroscope, and image processing. *IEEE Access*, 8, 129645–129667. doi: 10.1109/ACCESS.2020.3009226

[10] Bakker B., Zabłocki B., Baker A., Riethmeister V., Marx B., Iyer G., et al. (2021). A multi-stage, multi-feature machine learning approach to detect driver sleepiness in naturalistic road driving conditions. *IEEE Transactions on Intelligent Transportation Systems*, 23(5), 4791–4800. doi: 10.1109/TITS.2021.3090272

[11] Yogesh, R., Ritheesh, V., Reddy, S., & Rajan, R. G. (2022). Driver Drowsiness Detection and Alert System using YOLO. In *2022 International Conference on Innovative Computing*, *Intelligent Communication and Smart Electrical Systems (ICSES)* (pp. 1–6). IEEE.

[12] Gu H., Ji Q., & Zhu Z. (2002). Active facial tracking for fatigue detection. In *Sixth IEEE Workshop on Applications of Computer Vision*, *2002 (WACV 2002). Proceedings*. (pp. 137–142). IEEE.

[13] Fernández-Sotos P., García A. S., Vicente-Querol M. A., Lahera G., Rodriguez-Jimenez R., & Fernández-Caballero A. (2021). Validation of dynamic virtual faces for facial affect recognition. *Plos One*, 16(1), e0246001. doi: 10.1371/journal.pone.0246001 33493234 PMC7833130

[14] Valeriani D., & Poli R. (2019). Cyborg Astrobiologists: The Next Step in the Evolution of Exobiology. *Frontiers in Astronomy and Space Sciences*, 6, 75.

[15] Du G., Li T., Li C., Liu P. X., & Li D. (2020). Vision-based fatigue driving recognition method integrating heart rate and facial features. *IEEE Transactions on Intelligent Transportation Systems*, 22(5), 3089–3100. doi: 10.1109/TITS.2020.2979527

[16] Qiao, Y., Zeng, K., Xu, L., & Yin, X. (2016). A smartphone-based driver fatigue detection using fusion of multiple real-time facial features. In *2016 13th IEEE Annual Consumer Communications & Networking Conference (CCNC)* (pp. 230–235). IEEE.

[17] Wu, J., Chen, R., & Chen, W. (2019). Intelligent Adjusting System of Headlights based on Machine Vision. In *3rd International Conference on Computer Engineering*, *Information Science & Application Technology (ICCIA 2019)* (pp. 513–519). Atlantis Press.

[18] Yang R., Wang Z., Lin P.-S., Li X., Chen Y., Hsu P. P., et al. (2019). Safety effects of street lighting on roadway segments: Development of a crash modification function. *Traffic Injury Prevention*, 20(3), 296–302. doi: 10.1080/15389588.2019.1573317 30971143

[19] Waldner M., Müller N., & Bertram T. (2022). Energy-Efficient Illumination by Matrix Headlamps for Nighttime Automated Object Detection. *International Journal of Electrical and Computer Engineering Research*, 2(3), 8–14. doi: 10.53375/ijecer.2022.295

[20] Shen J., Li G., Yan W., Tao W., Xu G., Diao D., et al. (2018). Nighttime driving safety improvement via image enhancement for driver face detection. *IEEE Access*, 6, 45625–45634. doi: 10.1109/ACCESS.2018.2864629

[21] Mo Y., Han G., Zhang H., Xu X., & Qu W. (2019). Highlight-assisted nighttime vehicle detection using a multi-level fusion network and label hierarchy. *Neurocomputing*, 355, 13–23. doi: 10.1016/j.neucom.2019.04.005

[22] Schamm T., von Carlowitz C., & Zöllner J. M. (2010). On-road vehicle detection during dusk and at night. In *2010 IEEE Intelligent Vehicles Symposium* (pp. 418–423). IEEE. doi: 10.1109/IVS.2010.5548013

[23] Kim K.-H., Yum D.-H., Byeon D.-K., Kim D.-Y., & Lee D.-i. (2010). Improving driver’s visual field using estimation of curvature. In *ICCAS 2010* (pp. 728–731). IEEE.

[24] Akrout B., Mahdi W., “Yawning detection by the analysis of variational descriptor for monitoring driver drowsiness,” in *Proceedings of the 2016 International Image Processing*, *Applications and Systems (IPAS)*, pp. 1–5, 2016.

[25] Yang H., Liu L., Min W., Yang X., Xiong X., “Driver yawning detection based on subtle facial action recognition,” *IEEE Transactions on Multimedia*, vol. 23, pp. 572–583, 2020. doi: 10.1109/TMM.2020.2985536

[26] W. Zhang, J. Su, “Driver yawning detection based on long short term memory networks,” in *Proceedings of the 2017 IEEE Symposium Series on Computational Intelligence (SSCI)*, pp. 1-5, 2017.

[27] Anber S., Alsaggaf W., Shalash W., “A Hybrid Driver Fatigue and Distraction Detection Model Using AlexNet Based on Facial Features,” *Electronics*, vol. 11, no. 2, article 285, 2022. doi: 10.3390/electronics11020285

[28] P. Kielty, M. Sefidgar Dilmaghani, C. Ryan, J. Lemley, P. Corcoran, “Neuromorphic sensing for yawn detection in driver drowsiness,” in *Proceedings of the Fifteenth International Conference on Machine Vision (ICMV 2022)*, vol. 12701, pp. 287-294, 2023.

[29] Majeed F., Shafique U., Safran M., Alfarhood S., Ashraf I., “Detection of drowsiness among drivers using novel deep convolutional neural network model,” *Sensors*, vol. 23, no. 21, p. 8741, 2023. doi: 10.3390/s23218741 37960441 PMC10650052

[30] B.-T. Dong, H.-Y. Lin, “An On-board Monitoring System for Driving Fatigue and Distraction Detection,” in *Proceedings of the 2021 22nd IEEE International Conference on Industrial Technology (ICIT)*, pp. 850-855, 2021.

