# Supplementary material for: Illumination Intelligent Adaptation and Analysis Framework: A comprehensive solution for enhancing nighttime driving fatigue monitoring
Source: PLoS One. 2024 Aug 14;19(8):e0308201. doi: 10.1371/journal.pone.0308201 (PMC11324097; doi:10.1371/journal.pone.0308201)
Supplement: S1 Appendix — (PDF) [file pone.0308201.s001.pdf]

# Appendix: Mathematical Proofs

**Theorem 1** (Differential Optimization of the Fatigue Detection Model)**.** *In the optimization problem defined by (6), the solution set* $\{\alpha_i^*, \beta_k^*, U_{kl}^*, V_n^*, \gamma^*, \lambda^*\}$ *maximizes the differential accuracy of the fatigue assessment output* $Y_t$*. This is expressed as the derivative of* $Y_t$ *with respect to each parameter, ensuring that the rate of change in fatigue detection accuracy is optimized at every instant.*

$$\frac{dY_t}{d\theta} = 0, \quad \textit{for } \theta \in \{\alpha_i, \beta_k, U_{kl}, V_n, \gamma, \lambda\} \tag{1}$$

*where* $dY_t/d\theta$ *represents the partial derivative of the fatigue assessment output with respect to the model parameters.*

*Proof.* Consider the optimization problem defined in (6) with the objective to maximize the differential accuracy of the fatigue assessment output $Y_t$. Assume, for the sake of contradiction, that there is no unique optimal solution set $\{\alpha_i^*, \beta_k^*, U_{kl}^*, V_n^*, \gamma^*, \lambda^*\}$ that maximizes this differential accuracy.

From the differential perspective, the research considers the partial derivative of $Y_t$ with respect to each parameter $\theta \in \{\alpha_i, \beta_k, U_{kl}, V_n, \gamma, \lambda\}$:

$$\frac{dY_t}{d\theta} = \frac{d}{d\theta}\left(\gamma \cdot \sum_{m=1}^{Q} \delta_m \cdot F(X_{t-m}) + \lambda \cdot \sum_{n=1}^{R} \epsilon_n \cdot \text{Softmax}(V_n * P(X_{t-n}) + d_n)\right) \tag{2}$$

Given that $Y_t$ is a function of nonlinear transformations (as defined in (5)), the partial derivative $\frac{dY_t}{d\theta}$ involves computing the gradient through these nonlinear functions, including the ReLU and Softmax functions. However, due to the uniqueness property of derivatives in calculus, for each parameter set, there exists a unique gradient vector at any point in the parameter space.

To maximize $Y_t$ while minimizing prediction error, the research seeks the point where the gradient of $Y_t$ with respect to each parameter is zero, indicating a local extremum:

$$\nabla_\theta Y_t = \mathbf{0} \tag{3}$$

where $\nabla_\theta Y_t$ represents the gradient of $Y_t$ with respect to the parameters.

However, the uniqueness of the gradient at each point (due to the continuous differentiability of the nonlinear functions used) implies that there cannot be multiple parameter sets yielding the same gradient value of zero.This contradicts the initial assumption of non-uniqueness.

Therefore, there must exist a unique set of optimal solutions $\{\alpha_i^*, \beta_k^*, U_{kl}^*, V_n^*, \gamma^*, \lambda^*\}$ that maximizes the differential accuracy of $Y_t$.

This proof, leveraging advanced calculus, particularly the properties of continuous differentiability and gradient uniqueness, validates the uniqueness of the solution set for the optimization problem.

**Theorem 2** (Convergence Analysis through Integral Calculus)**.** *The boundedness and convergence of the IIAAF model, as defined in (6), can be analyzed through integral calculus. The cumulative effect of incremental changes in model parameters on the*

*fatigue assessment accuracy is bounded, ensuring the model's stability over continuous operational periods.*

$$\int_{\alpha_i}^{\alpha_i^*} \int_{\beta_k}^{\beta_k^*} \cdots \int_{\lambda}^{\lambda^*} \frac{\partial Y_t}{\partial \theta} d\theta \textit{ is finite and bounded} \tag{4}$$

*where* $\frac{\partial Y_t}{\partial \theta}$ *represents the partial derivative of* $Y_t$ *with respect to each parameter* $\theta$*.*

*Proof.* Assume for contradiction in the optimization formulation (6) that the set of optimal solutions is either unbounded or does not guarantee convergence.

To address boundedness, suppose that the optimal solution set is unbounded. Consider the parameter $\alpha_i$ without loss of generality. If $\alpha_i$ is unbounded, it implies that $Y_t$ can grow infinitely for increasing values of $\alpha_i$. However, considering the bounded nature of activation functions (ReLU and Softmax) used in $Y_t$ as defined in (5), there exists an upper limit to the range of $Y_t$. Hence, there must exist a bound beyond which any increase in $\alpha_i$ does not significantly affect $Y_t$, contradicting the assumption of unboundedness. This contradiction implies that the optimal solution set is bounded.

For convergence, the research uses the concept of Cauchy sequences. Suppose the optimal solution set does not guarantee convergence. Then, there exists a Cauchy sequence of parameter sets $\{\theta_n\}$ such that $\theta_n \to \theta^*$, but $Y_t(\theta_n)$ does not converge to $Y_t(\theta^*)$. This non-convergence contradicts the continuous nature of $Y_t$, given its dependence on continuous activation functions and model parameters. Hence, every Cauchy sequence of parameter sets must converge in $Y_t$, ensuring the convergence of the solution set.

Furthermore, the research introduces an integral calculus approach to reinforce the boundedness and convergence argument:

$$\int_{\alpha_i}^{\alpha_i^*} \int_{\beta_k}^{\beta_k^*} \cdots \int_{\lambda}^{\lambda^*} \left| \frac{\partial^2 Y_t}{\partial \theta^2} \right| d\theta \text{ is finite} \tag{5}$$

where $\frac{\partial^2 Y_t}{\partial \theta^2}$ is the second-order partial derivative of $Y_t$ with respect to $\theta$. The finiteness of this integral indicates that even with incremental changes in parameters, the rate of change of $Y_t$ is bounded, further confirming the boundedness and convergence of the optimal solution set.

In conclusion, the assumption of unboundedness or non-convergence is invalid; hence, the set of optimal solutions in the optimization formula (6) must be both bounded and guarantee convergence. This completes the proof by contradiction and integral calculus.

**Theorem 3** (Optimized Dynamic Illumination Adjustment)**.** *For the real-time brightness adjustment formula (10), an optimal parameter set* $\{\theta_p, \eta_{pq}, \xi_p, \gamma_r, \omega_{rs}, \zeta_r, \lambda_u, \mu_{uv}, \rho_u\}$ *exists, which maximizes the overall illumination adjustment of each pixel in image frames* $I_{xy,t}$*. The optimization condition is represented by the following equation:*

$$\frac{\partial}{\partial \theta} \left( \sum_{t=1}^{T} \sum_{x,y} A(I_{xy,t}) \right) = 0, \quad \forall \theta \in \{\theta_p, \eta_{pq}, \xi_p, \gamma_r, \omega_{rs}, \zeta_r, \lambda_u, \mu_{uv}, \rho_u\} \tag{6}$$

*where* $\frac{\partial}{\partial \theta}$ *indicates the partial derivative with respect to each parameter* $\theta$*, ensuring optimal brightness and contrast adjustment for enhanced visibility and image quality.*

*Proof.* Given the real-time brightness adjustment optimization formula (10), the research seeks to prove the existence of an optimal parameter set $\{\theta_p, \eta_{pq}, \xi_p, \gamma_r, \omega_{rs}, \zeta_r, \lambda_u, \mu_{uv}, \rho_u\}$ that maximizes the function $A(I_{xy}, t)$, which adjusts brightness and contrast for each pixel.

The optimization problem is formulated as:

$$\max_{\theta_p,\eta_{pq},\xi_p,\gamma_r,\omega_{rs},\zeta_r,\lambda_u,\mu_{uv},\rho_u} \sum_{t=1}^{T} \sum_{x,y} A(I_{xy,t}) \tag{7}$$

To find the optimal parameters, the research computes the gradient of the objective function with respect to each parameter and sets it to zero. This yields a system of nonlinear equations:

$$\frac{\partial}{\partial\theta}\left(\sum_{t=1}^{T}\sum_{x,y} A(I_{xy,t})\right) = 0, \quad \forall\theta \in \{\theta_p, \eta_{pq}, \xi_p, \gamma_r, \omega_{rs}, \zeta_r, \lambda_u, \mu_{uv}, \rho_u\} \tag{8}$$

Solving this system requires iterative numerical methods, such as gradient ascent, due to its nonlinearity and high dimensionality. Each iteration updates the parameter values in the direction that increases the sum of $A(I_{xy}, t)$ across all pixels and frames.

Furthermore, the research introduces a constraint to ensure the boundedness of the solution:

$$\sum_{\theta}\left|\frac{\partial^2}{\partial\theta^2}\left(\sum_{t=1}^{T}\sum_{x,y} A(I_{xy,t})\right)\right| < \infty \tag{9}$$

where $\frac{\partial^2}{\partial\theta^2}$ represents the second-order partial derivative, ensuring that the adjustment does not lead to extreme values that could distort the image.

Through this approach, the research finds an optimal set of parameters that not only maximize the objective function but also respect the physical constraints of image processing, ensuring improved image quality and visibility of facial features.

In conclusion, the existence of an optimal parameter set for the per-pixel dynamic light adjustment is validated, meeting both the optimization criteria and practical constraints.

**Corollary 1** (Real-Time Adjustment and Environmental Adaptability)**.** *In the formulation of Problem 3 (11), the per-pixel dynamic light adjustment technique, through the implementation of the optimization model (10), demonstrates high adaptability to rapidly changing lighting conditions. Specifically, the technique can adapt to fluctuations in environmental lighting $\Delta L_t$ through the adjustment function $A(I_{xy}, t)$, expressed as:*

$$A(I_{xy}, t + \Delta t) = A(I_{xy}, t) + \frac{\partial A}{\partial L_t}\Delta L_t \tag{10}$$

*where $\Delta L_t$ represents the change in lighting, such as streetlights flickering or headlights from oncoming vehicles. This adaptability optimizes the accuracy and reliability of driver fatigue detection.*

*Proof.* Let $A(I_{xy}, t)$ be the real-time brightness adjustment model, defined by (10). Consider the impact of lighting changes $\Delta L_t$ on $A(I_{xy}, t)$.

The research assumes that $A(I_{xy}, t)$ can adapt to lighting changes. To prove this, it needs to be shown that $A(I_{xy}, t + \Delta t)$ can adapt to $\Delta L_t$.

Considering the impact of lighting change $\Delta L_t$, it can be represented as $I_{xy,t+\Delta t} = I_{xy,t} + \Delta L_t$. According to (9) and (10), the research can write out $A(I_{xy}, t + \Delta t)$:

$$A(I_{xy}, t + \Delta t) = \sum_{r=1}^{R} \gamma_r \cdot \text{Sigmoid}\left(\sum_{s=1}^{S} \omega_{rs} \cdot L(I_{xy,t+\Delta t-s}) + \zeta_r\right) \tag{11}$$

Since $L(I_{xy}, t)$ is an adjustment to brightness, the research can approximate $L(I_{xy,t+\Delta t-s})$ as $L(I_{xy,t-s}) + \Delta L_t$. Thus, it can approximate $A(I_{xy}, t + \Delta t)$ as:

$$A(I_{xy}, t + \Delta t) \approx A(I_{xy}, t) + \sum_{r=1}^{R} \gamma_r \cdot \text{Sigmoid}'(\cdots) \cdot \Delta L_t + \cdots \tag{12}$$

Where $\text{Sigmoid}'(\cdots)$ represents the derivative of the Sigmoid function. This indicates that the change in $A(I_{xy}, t + \Delta t)$ is proportional to $\Delta L_t$, i.e., $A(I_{xy}, t)$ can adapt to changes in lighting.

Therefore, the per-pixel dynamic light adjustment technique indeed can adapt to rapid changes in environmental lighting, especially capable of adjusting the function $A(I_{xy}, t)$ according to changes in lighting, thereby optimizing the accuracy and reliability of driver fatigue detection.

**Corollary 2** (Environmental Light Adaptability)**.** *In the optimization formula (18), the light variation feature learning system exhibits significant adaptability to changes in environmental lighting. This adaptability is not limited to recognizing current lighting conditions but also extends to predicting and adapting to future lighting changes. Specifically, through the model $L(I_{xy}, t)$, the system can predict changes in lighting at a future time $t + \Delta t$, expressed as:*

$$L(I_{xy}, t + \Delta t) \approx L(I_{xy}, t) + \frac{\partial L}{\partial t} \Delta t \tag{13}$$

*where $\frac{\partial L}{\partial t}$ represents the time derivative, reflecting the dynamic characteristics of light changes.*

*Proof.* First, let's consider the definition of the model $L(I_{xy}, t)$. According to (17):

$$L(I_{xy}, t) = \sum_{r=1}^{R} \mu_r \cdot \left( \text{Sigmoid} \left( \sum_{s=1}^{S} \nu_{rs} \cdot P(I_{xy,t-s}) + \xi_r \right) \right) \tag{14}$$

Where $P(I_{xy}, t)$ is the light variation prediction model defined by time series analysis and feature extraction. The research needs to prove the model's adaptability to time changes.

Assume there are lighting changes $\Delta L_t$ affecting the image frame $I_{xy,t}$. According to the definitions of $G(I_{xy}, t)$ and $T(I_{xy}, t)$, the research can represent $P(I_{xy}, t + \Delta t)$ and $L(I_{xy}, t + \Delta t)$.

Using a Taylor series expansion of $L(I_{xy}, t + \Delta t)$ to the first order:

$$L(I_{xy}, t + \Delta t) \approx L(I_{xy}, t) + \frac{\partial L}{\partial t} \Delta t \tag{15}$$

Where $\frac{\partial L}{\partial t}$ is the partial derivative of $L(I_{xy}, t)$ with respect to time $t$. The research needs to show that $\frac{\partial L}{\partial t}$ reflects the dynamic characteristics of light changes.

Since $L(I_{xy}, t)$ is based on $P(I_{xy}, t)$, the research further considers the changes in $P(I_{xy}, t)$. According to the definition of $P(I_{xy}, t)$, it can represent $\frac{\partial P}{\partial t}$. Then, using the chain rule and $\frac{\partial P}{\partial t}$, it calculates $\frac{\partial L}{\partial t}$:

$$\frac{\partial L}{\partial t} = \sum_{r=1}^{R} \mu_r \cdot \text{Sigmoid}'(\cdots) \cdot \frac{\partial P}{\partial t} \tag{16}$$

Where $\text{Sigmoid}'(\cdots)$ represents the derivative of the Sigmoid function.

Combining the definition of $\frac{\partial P}{\partial t}$ and the response of $P(I_{xy}, t)$ to lighting changes, the research can see that $\frac{\partial L}{\partial t}$ reflects the dynamic characteristics of light changes, thereby proving the environmental adaptability of the light variation feature learning system.

**Corollary 3** (Predictive Accuracy and Stability)**.** *The comprehensive predictive model $P(I_{xy}, t)$ based on the light variation feature learning system not only accurately predicts short-term changes in lighting but also exhibits excellent long-term stability. This effect is attributed to the model's deep network structure and integrated time series analysis, thus ensuring the stability and accuracy of fatigue detection in complex nighttime driving environments.*

*Proof.* First, the research considers the definition of the model $P(I_{xy}, t)$. According to (16), it has:

$$P(I_{xy}, t) = \sum_{m=1}^{M} \gamma_m \cdot \text{Tanh} \left( \sum_{n=1}^{N} \delta_{mn} \cdot T(I_{xy}, t-n) + \epsilon_m \right) \tag{17}$$

Here, $T(I_{xy}, t)$ is a time series analysis model based on $G(I_{xy}, t)$.

To prove predictive accuracy, the research first considers the model's response to current lighting conditions. As $G(I_{xy}, t)$ is based on current and historical image data, it can be assumed that it accurately extracts features under current lighting conditions. Therefore, $T(I_{xy}, t)$ and $P(I_{xy}, t)$ can also accurately reflect current lighting conditions.

Regarding long-term stability, the research considers the model's response to time series data. Since $P(I_{xy}, t)$ combines $T(I_{xy}, t)$ and $G(I_{xy}, t)$, it can capture and predict trends in lighting conditions. Mathematical expectation and variance are used to quantify this prediction's stability.

Let $E[P(I_{xy}, t)]$ and $Var[P(I_{xy}, t)]$ represent the expectation and variance of $P(I_{xy}, t)$, respectively. As $P(I_{xy}, t)$ combines historical and current data, it can be assumed that over time, $E[P(I_{xy}, t)]$ tends to stabilize and $Var[P(I_{xy}, t)]$ decreases. The research further considers the contribution of the deep network structure to stability. As deep networks can capture more complex features, they can better adapt to environmental changes, thereby improving prediction stability.

Ultimately, by combining time series analysis and a deep network structure, $P(I_{xy}, t)$ can provide accurate and stable predictions in complex nighttime driving environments, thereby ensuring the stability and accuracy of fatigue detection.
